# Materials Design by Constructing Phase Diagrams for Defects

**DOI:** 10.1002/adma.202402191

**Published:** 2024-11-17

**Authors:** Xuyang Zhou, Prince Mathews, Benjamin Berkels, Wassilios Delis, Saba Saood, Amel Shamseldeen Ali Alhassan, Philipp Keuter, Jochen M. Schneider, Sandra Korte‐Kerzel, Stefanie Sandlöbes‐Haut, Dierk Raabe, Jörg Neugebauer, Gerhard Dehm, Tilmann Hickel, Christina Scheu, Siyuan Zhang

**Affiliations:** ^1^ Max Planck Institute for Sustainable Materials Max‐Planck‐Straße 1 40237 Düsseldorf Germany; ^2^ Aachen Institute for Advanced Study in Computational Engineering Science (AICES) RWTH Aachen University Schinkelstraße 2 52062 Aachen Germany; ^3^ Insitute for Physical Metallurgy and Materials Physics RWTH Aachen 52074 Aachen Germany; ^4^ Materials Chemistry RWTH Aachen University Kopernikusstraße 10 52074 Aachen Germany; ^5^ Federal Institute for Materials Research and Testing (BAM) Richard‐Willstätter‐Straße 11 12489 Berlin Germany

**Keywords:** automatic pattern recognition, defect phase diagram, density functional theory, grain boundary complexion, transmission electron microscopy

## Abstract

Phase transformations and crystallographic defects are two essential tools to drive innovations in materials. Bulk materials design via tuning chemical compositions is systematized using phase diagrams. It is shown here that the same thermodynamic concept can be applied to manipulate the chemistry at defects. Grain boundaries in Mg–Ga system are chosen as a model system, because Ga segregates to the boundaries, while simultaneously improving the strength and ductility of Mg alloys. To reveal the role of grain boundaries, correlated atomic‐scale characterization and simulation to scope and build phase diagrams for defects are presented. The discovery is enabled by triggering phase transformations of individual grain boundaries through local alloying, and sequentially imaging the structural and chemical changes using atomic‐resolution scanning transmission electron microscopy. Ab initio simulations determined the thermodynamic stability of grain boundary phases, and found out that increasing Ga content enhances grain boundary cohesion, relating to improved ductility. The methodology to trigger, trace, and simulate defect transformation at atomic resolution enables a systematic development of defect phase diagrams, providing a valuable tool to utilize chemical complexity and phase transformations at defects.

## Graphical abstract

A systematic way to develop defect phase diagrams is proposed for advanced materials design. Phase transformation of a Mg grain boundary is triggered by Ga implantation on a microscopy sample. Transformations of structural units and chemical ordering at the same grain boundary are experimentally traced at atomic resolution and modeled by ab initio simulations to construct the defect phase diagram.

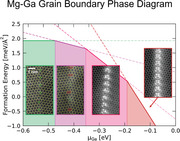

## 1 Introduction

Materials development is the backbone of the advancement of human civilization. Mastering multi‐phase materials and their associated transformations allows for customized applications in infrastructure, transportation,^[^ ^1^ ^]^ energy,^[^ ^2^ ^]^ and medical devices.^[^ ^3^ ^]^ As a well‐organized, systematic thermodynamic approach to design materials, phase diagrams serve as a crucial instrument for comprehending the effects of variables such as temperature, pressure, and chemical composition on phases and properties.^[^ ^4^ ^]^

While phase diagrams predict the constituent phases and their coexistence in materials, they do not account for crystallographic defects which however control many materials properties. Examples are dislocations and grain boundaries (GBs) which determine many mechanical,^[^ ^5^, ^6^ ^]^ transport^[^ ^7^ ^]^ and functional properties.^[^ ^8^ ^]^ In addition to the local structural distortions that are characteristic of defects, their chemical composition can also deviate significantly from that of the surrounding bulk phase. For example, solute elements can segregate around dislocations (Cottrell atmospheres^[^ ^9^ ^]^), stacking faults (Suzuki effect^[^ ^13^, ^14^ ^]^), and GBs.^[^ ^15^, ^16^, ^17^, ^18^, ^19^, ^20^, ^21^, ^22^, ^23^, ^24^, ^25^, ^26^ ^]^ Such effects have been increasingly employed, as materials design goes through a paradigm shift to exploit rather than avoid the chemical complexity around defects. Such confined and low‐dimensional chemical states have been termed as “low‐dimensional phases,”^[^ ^27^, ^28^, ^29^ ^]^ “complexions,”^[^ ^23^, ^30^, ^31^, ^32^, ^33^, ^34^ ^]^ or “defect phases”^[^ ^19^, ^35^, ^36^, ^37^, ^38^ ^]^ to differentiate them from bulk phases.

GBs separate regions of individual single‐crystalline grains and can control functional properties such as electrical resistivity,^[^ ^8^, ^39^, ^40^, ^41^ ^]^ magnetic coercivity,^[^ ^42^ ^]^ as well as mechanical strength and ductility.^[^ ^5^, ^32^, ^43^, ^44^, ^45^, ^46^, ^47^, ^48^ ^]^ The thermodynamic theory on GB phase transformations^[^ ^27^, ^49^, ^50^, ^51^ ^]^ has been developed further in the last decade by Frolov and Mishin.^[^ ^52^, ^53^ ^]^ It has been recently revealed that different defect phases can coexist at GBs even in elemental metals with only a single bulk phase, such as Cu.^[^ ^54^, ^55^, ^56^ ^]^ Moreover, the introduction of alloying elements can be utilized to control these defect phases, their transformations and in turn the material's properties, opening new doors to evolve materials design.^[^ ^20^, ^47^ ^]^ Different defect systems have demonstrated phase transformations. For example, on the mesoscopic scale, GB faceting transformations were observed in Cu GBs, triggered by adding Bi^[^ ^57^ ^]^ or Ag.^[^ ^58^ ^]^ In another case, segregation of alloying elements or impurities can lead to embrittlement of the metal through the formation of a liquid GB phase,^[^ ^24^, ^32^, ^59^ ^]^ e.g. Ga embrittlement of Al alloys.^[^ ^60^, ^61^ ^]^

Ga solutes have been found beneficial in other alloys, e.g. Mg.^[^ ^62^, ^63^, ^64^, ^65^, ^66^, ^67^ ^]^ For materials design, the Mg–Ga phase diagram has been developed to predict important parameters, such as the solubility of Ga within Mg alloys.^[^ ^68^ ^]^ Nevertheless, the mechanical properties are largely determined by defects, including GBs, but there is little known about the distribution of Ga within these defects, where the thermodynamics is very different from the bulk phases.

In this article, we use Mg–Ga GBs as a model system to develop defect phase diagrams. After introducing the bulk mechanical properties of Mg–Ga, we present a local alloying approach to tune the chemical potential of Ga at GBs and construct a defect phase diagram. For this, we employ a direct correlation between the defect phase transformations observed experimentally at atomic resolution using scanning transmission electron microscopy (STEM) and simulations using density functional theory (DFT). The experimental observation was accomplished by following the structural transformation of the same GB after 1) local alloying of Ga via focused ion beam (FIB) milling and 2) long‐term natural aging to approach thermal equilibrium. The methodology can be employed to populate defect phase diagrams for many more nanoscopic defects crucial to the design of modern materials.

## 2 Results and Discussion

### 2.1 Mechanical Properties of Mg–Ga Alloys

**Figure 1:**
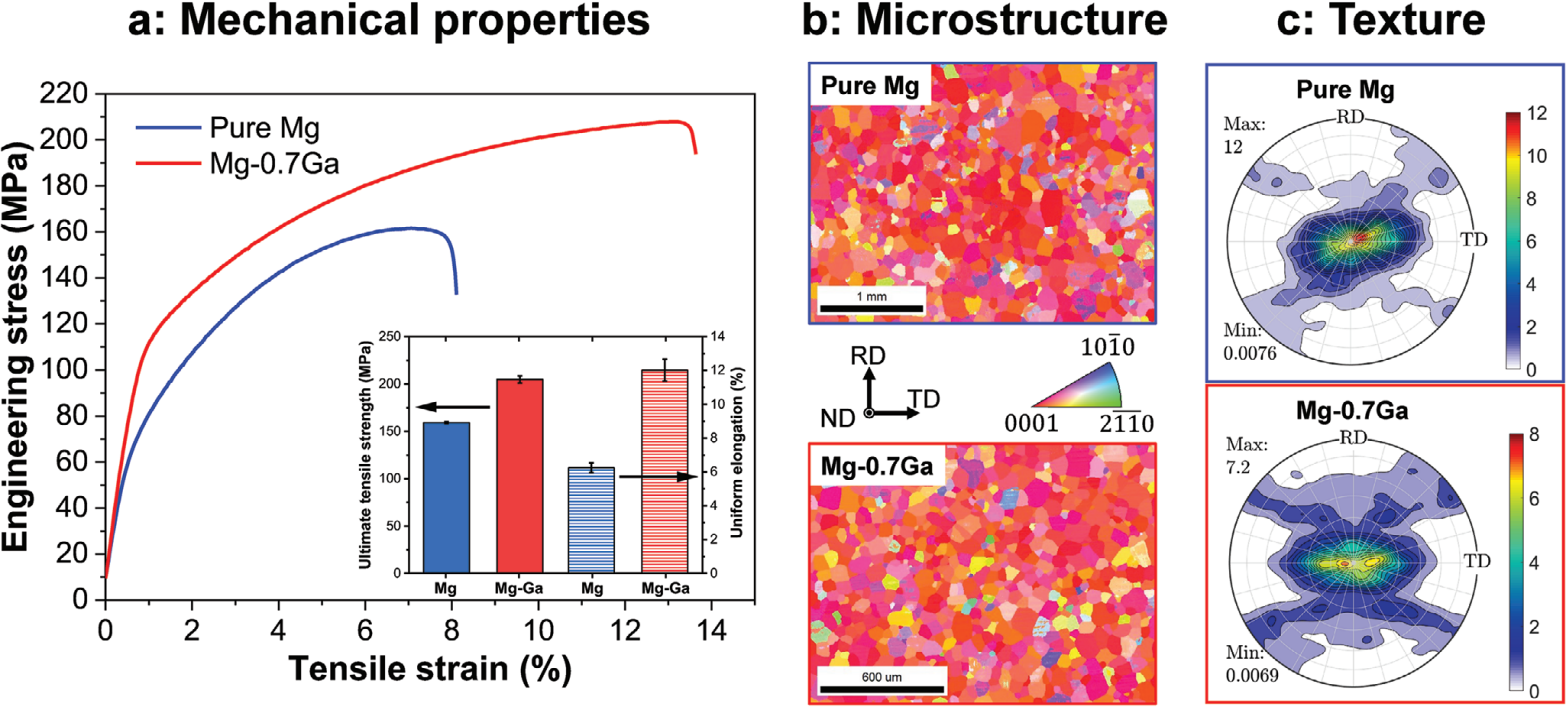
Microstructure‐property relationship of model Mg–Ga alloys. a) Stress‐strain curves showing the improved tensile strength and ductility of Mg‐0.7at.%Ga with respect to pure Mg. The inset shows the statistics on the ultimate tensile strength and uniform elongation. b) Electron backscatter diffraction (EBSD) and c) X‐ray (0002) pole figure maps showing the weakened basal‐plane texture of Mg‐0.7at.%Ga compared to pure Mg.

Alloying Ga into Mg has been demonstrated to be beneficial with respect to both strength and ductility of the alloys.^[^ ^62^, ^63^, ^64^, ^65^, ^66^, ^67^ ^]^ As shown in **Figure** **1**a, adding just 0.7 at.% Ga to Mg increases the ultimate tensile strength from 159 MPa to 205 MPa, while almost doubling the uniform elongation from 6.2% to 12.0%. Note that 0.7 at.% Ga corresponds to its solubility limit in Mg at room temperature, and hence both samples are free of precipitates. Furthermore, both samples have a large grain size after recrystallization (Figure 1b), on the order of 100 µm, which gives little Hall‐Petch strengthening. Therefore, the increase in tensile strength can be mostly attributed to strengthening from Ga solutes. ^[^ ^63^, ^65^, ^66^, ^67^ ^]^

As was shown in many Mg alloys,^[^ ^69^ ^]^ the significant improvement in tensile ductility is linked to changes in their crystallographic texture, which can be effectively controlled via GB engineering.^[^ ^70^ ^]^ In rolled Mg, a sharp basal‐type texture is commonly observed. In this orientation, the Schmid factor for basal slip is zero, and hence the activation of basal slip is very unlikely. As activation of other slip systems in Mg have orders of magnitude higher critical resolved shear stresses, early failure along macroscopic shear bands is observed.^[^ ^71^ ^]^ Mg alloys with weaker basal‐type texture contain more grains with non‐zero Schmid factors for basal slip, which contribute greatly to improved ductility. As shown in Figure 1c, the addition of Ga weakens the basal‐type texture (from 12 multiples of a random distribution in pure Mg to 7.2 multiples in Mg‐0.7 at.% Ga), suggesting a reduction of the abnormal grain growth of grains with [0001] orientation resulting from the interplay between Ga and Mg GBs.

To understand the [0001] oriented grains and the role of Ga at their boundaries, [0001] tilt GBs need to be characterized down to the atomic scale. Recently, Ga atoms were found to segregate at Mg GBs,^[^ ^72^ ^]^ although it is unclear whether they form specific structures to be treated as GB phases. To resolve the atomistic structure at GBs, a high symmetry [0001] tilt GB was selected from a Mg thin film with a sharp basal‐type texture (Figure S1, Supporting Information).^[^ ^72^ ^]^ Compared to the bulk tensile samples, textured Mg thin films contain GBs with pure tilt character, which facilitate imaging at atomic resolution. By monitoring Ga incorporation into a single GB at atomic resolution, we could evidence segregation of Ga to the GB, triggering GB phase transformation of different structures and chemical ordering.

### 2.2 GB Phase Transformations Triggered by Local Alloying

**Figure 2:**
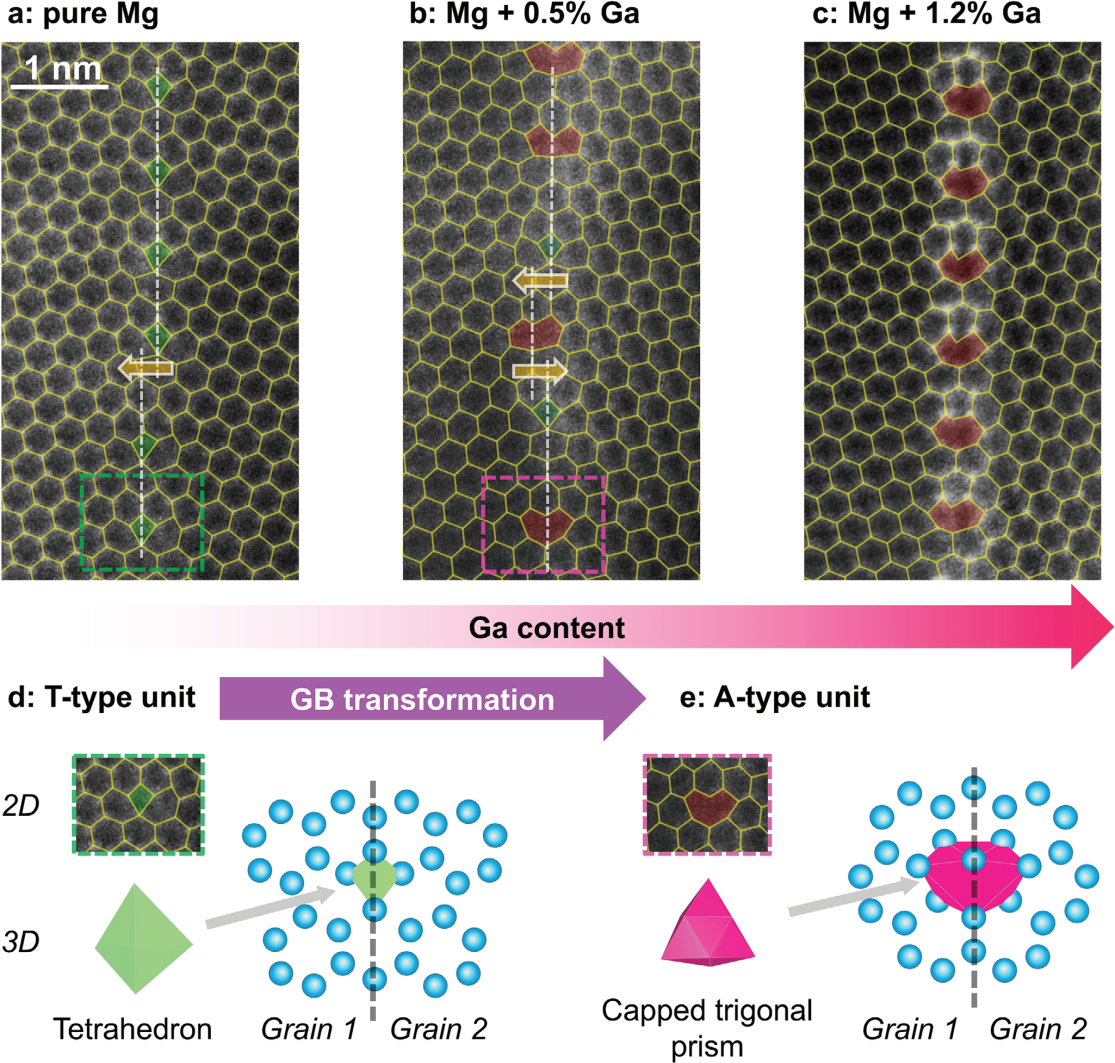
Experimental observation of a GB phase transformation in Mg by local alloying of Ga. a‐c) High angle annular dark field (HAADF)‐STEM images of a Σ7 GB in pure Mg, (b) Mg‐0.5at.%Ga and (c) Mg‐1.2at.%Ga. The images are overlaid with polygon grids to highlight the GB structural units (Section 4, Automatic Pattern Recognition), while raw experimental images are presented in Figure S4 (Supporting Information). The dashed lines in (a) and (b) highlight the GB planes and the arrows point to shifts in the GB plane. d) Atomistic structures of the T‐type structural unit (green tetrahedron) and e) the A‐type structural unit (pink capped trigonal prism) in 2D and 3D.

To study GB phases on the atomic scale, we selected symmetric GBs in Mg along the [0001] tilt axis, which can adopt a variety of atomistic structures. In particular, the Σ7 [0001]$\left\{3\bar{1}\bar{2}0\right\}$ GBs (abbreviated as Σ7 GBs) have a misorientation angle of ≈22°,^[^ ^72^ ^]^ and two types of structural units, T‐type and A‐type.^[^ ^73^ ^]^ Moreover, Σ7 GBs can be also considered as arrays of edge dislocations, with each T‐type or A‐type structural unit viewed as a dislocation core with a Burgers vector of $\frac{1}{3}\left\langle 2\bar{1}\bar{1}0\right\rangle$ (Figure S2, Supporting Information) and periodically spaced at 0.85 nm. Experimentally, we have identified one Σ7 GB in Mg‐based on 4D‐STEM mapping (see Figure S1, Supporting Information). As shown in **Figure** **2**a, the Σ7 GB is atomically resolved by STEM imaging, where T‐type structural units are found along the GB plane. Such T‐type units are common structural units to build GBs,^[^ ^74^, ^75^, ^76^ ^]^ which have a tetrahedron shape in 3D, as shown in Figure 2d. Beside the T‐type units, GB steps (e.g., arrow in Figure 2a) are also found along the GB, causing a shift in the GB plane.

We then applied local alloying by Ga^+^ implantation and subsequent diffusion to the Mg sample and returned to the same Σ7 GB for high resolution imaging (details in Section 4, Local Alloying to Trigger Defect Phase Transformation; Figure S3, Supporting Information). As shown in Figure S4b,c (Supporting Information), the GB areas show a higher atomic number (Z) contrast than the surrounding grains, demonstrating the tendency of Ga atoms to segregate at GBs.^[^ ^72^ ^]^ Closer examination reveals that after local alloying of 0.5 at.% Ga, some GB structural units have been transformed to A‐type units (Figure 2b), and after local alloying of 1.2 at.% Ga, the GB has been fully transformed into A‐type units (Figure 2c). The A‐type unit has a larger core structure and a capped trigonal prism shape in 3D (Figure 2e), where the same Burgers vector $\frac{1}{3}\left\langle 2\bar{1}\bar{1}0\right\rangle$ can be constructed (Figure S2b, Supporting Information).

In pure Mg, the formation energies of Σ7 GBs composed of T‐type and A‐type structural units are evaluated by DFT calculations as 0.309 and 0.311 J m^−2^, respectively. The experimentally observed T‐type unit is indeed the ground state configuration for Mg Σ7 GB, although the formation energy for A‐type Σ7 GB is only marginally higher, as was reported in previous DFT calculations on Mg^[^ ^77^ ^]^ and ZnO^[^ ^78^ ^]^ Σ7 GBs. To understand the structural transformation of the GB, we simulated the local alloying by replacing one Mg column in the T‐type unit by Ga and studying their structural relaxation by DFT. The atomic sites for the structural units are labeled in alphabetical order with respect to the increasing distance to the GB plane, as shown in Figure S2a,b (Supporting Information). The T‐type unit with the **b2** site occupied by Ga atoms spontaneously relaxes into an A‐type unit with Ga atoms occupying the **a1** site, as can be seen in Figure S5a (Supporting Information). A similar structural transformation sequence with Ga occupying a neighboring site is presented in Figure S5b (Supporting Information). In each relaxation step, the identical DFT structure is viewed from two perspectives, matching to the T‐type (filled green shape) and A‐type (open pink shape) structural units. As the GB structure relaxes, the distortion of the T‐type unit increases, while the A‐type unit becomes more symmetric. This visualization shows the shift of the local symmetric structure from the T‐type to the A‐type structural unit, completing the GB phase transformation. Without Ga atoms, Mg Σ7 GBs with T‐type structural units are confirmed as remaining stable during DFT structural relaxation (Figure S5c, Supporting Information). Although A‐type structural units are also stable during relaxation (Figure S5c, Supporting Information), the formation energy is slightly higher than T‐type.

To trace the GB phase transformation from STEM images, we have developed automatic pattern recognition to classify experimental GB structural units into T‐type and A‐type, as overlaid in Figure 2a–c. As schematically shown in Figure S6 (Supporting Information) and detailed in Section 4, Automatic Pattern Recognition, DFT structures serve as inputs to locate GB structural units in the STEM images. Then the positions of the atomic columns are labeled and compared with the DFT structures to reach a decision on a better match to T‐type or A‐type units. Moreover, it is observed from Figure S5 (Supporting Information) that after the structural transformation, the GB plane is shifted by $\frac{1}{6}\left\langle 2\bar{1}\bar{1}0\right\rangle$, half of the Burgers vector. This is experimentally captured between the middle A unit and its adjacent T units, as indicated by arrows in Figure 2b.

### 2.3 GB Phase Transformation with Different Chemical Ordering

**Figure 3:**
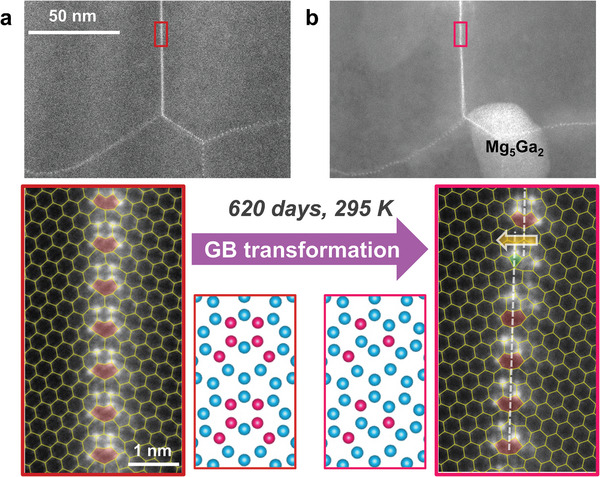
Transformation of chemically‐ordered GB phases. HAADF‐STEM images of the same Σ7 GB a) 1 day and b) 620 days after Ga^+^ beam thinning. The long storage time enabled Ga atoms on the surface to form bulk Mg_5_Ga_2_ precipitates, while the ordered 6‐Ga unit (a) transforms to a differently ordered 3‐Ga unit (b). The Ga atoms in the atomic configurations are highlighted in pink, with their atomic sites labeled. The A‐type structural units are highlighted by pink‐capped trigonal prisms. The corresponding STEM images without overlaid grids are presented in Figure S7 (Supporting Information).

With increasing amount of local alloying, chemical ordering of Ga atoms starts to appear, as shown by the brighter atomic columns at the GB (Figure S4c, Supporting Information). The chemical ordering is also reproduced in a TEM sample processed and thinned down completely with FIB of Ga^+^, as an attempt to introduce as much Ga as possible into the sample, and hence the GBs. Ga segregation to the GBs is evidently shown in **Figure** **3**a, and it is clear that the Ga‐decorated Σ7 GB is composed of A‐type structural units. Moreover, there is a clear pattern of Ga segregation to pairs of **b**, **c**, and **e** sites, in total six atomic columns for each structural unit.

After 620 days of storage in a desiccator, the same GB underwent a transformation to another ordered phase, as shown in Figure 3b. Instead of six, only three bright Ga columns remained in each structural unit. The Ga atoms stay on the **b**, **c**, and **e** sites, except that they are no longer present in pairs. In the upper part of the high‐resolution image in Figure 3b, the **b1** site is occupied by Ga along with the **c2** and **e2** sites on the right. Likewise, the lower part of the image shows the mirrored occupation on **b2**, **c1** and **e1** sites. Except for a structural unit at the disconnection, all other units remain as A‐type. Energy dispersive X‐ray spectroscopy (EDS) reveals a Ga content of 0.7 at.% inside the Mg grains, which increases significantly at the Σ7 GB (Figure S7g,h, Supporting Information). For this GB, the Gibbsian interfacial excess (shaded area in Figure S7h, Supporting Information) is 6.95 Ga atoms nm^−2^, corresponding to a coverage of 3 Ga atoms per structural unit. As there are exactly three bright columns in each structural unit, the ordered phase is simulated using full Ga occupancy at the corresponding sites. Likewise, the ordered phase with six bright columns is also simulated using full Ga occupancy, as the Gibbsian interfacial excess is estimated to be close to 6 Ga atoms per structural unit (Note S1, Supporting Information).

### 2.4 Construction of the GB Defect Phase Diagram

**Figure 4:**
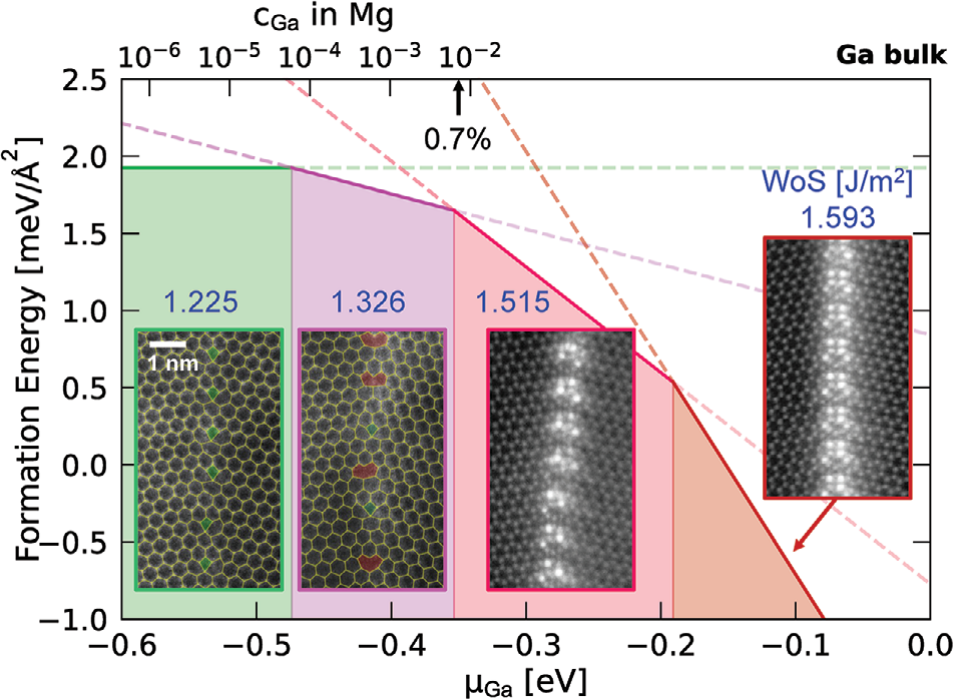
Construction of a defect phase diagram from observed phase transformations. The calculated Mg Σ7 GB phase diagram using DFT and experimentally‐observed GB structures. The bottom axis µ_Ga_ is the chemical potential of Ga with respect to the bulk Ga phase. The top axis provides the corresponding dilute Ga concentration of up to 1% in the Mg solid solution at 300 K. The arrow points to the measured $c_{\mathrm{Ga}}=0.7\%$ in the solid solution. The values on top of the experimental images refer to the DFT‐calculated work of separation (WoS) of the four GB phases.

Having identified the structural transformation (from T‐type to A‐type) of Mg Σ7 GB triggered by local alloying of Ga, as well as the transformation to different ordering of Ga, we investigate the thermodynamic stability of the observed GB phases in the framework of a defect phase diagram.^[^ ^38^ ^]^. As shown in **Figure** **4**, four ordered structures of Mg Σ7 GBs were simulated by DFT calculations, including respectively 0, 1, 3, and 6 Ga atoms in the supercells. For a single bulk composition, the elements partition differently among the bulk phase and various types of defects. Therefore, as the numbers of Mg and Ga atoms, *N*_Mg_ and *N*_Ga_, differ in each phase, the formation energy *E*_f_ is a function of the chemical potentials, µ_Mg_ and µ_Ga_, according to Equation (1), where *E*_GB_ is the total energy of the supercells, and *A*_GB_ the GB area.

(1) $$E_{f}=\frac{E_{\mathrm{GB}}-N_{\mathrm{Mg}}\mu_{\mathrm{Mg}}-N_{\mathrm{Ga}}\mu_{\mathrm{Ga}}}{2A_{\mathrm{GB}}}$$

With the elemental partition reaching local thermodynamic equilibrium, the chemical potential becomes constant across the bulk and the defect phases, and can therefore be used as the universal state variable to construct defect phase diagrams.^[^ ^38^ ^]^ As a reference state, µ_Mg_ and µ_Ga_ are set to zero when they are in equilibrium with a reservoir of Mg and Ga atoms in their bulk phases, respectively. As the entire sample is made of Mg, it fulfills the Mg‐rich condition µ_Mg_ = 0 eV. On the other hand, the Ga‐rich condition µ_Ga_ = 0 eV is only achieved when a layer of Ga is formed on the sample surface, e.g. after Ga FIB milling.

The formation energy of the four modeled structures is plotted as a function of the chemical potential (Figure 4) and they are predicted to be stable in different ranges of µ_Ga_. This is because each line of formation energy has a unique slope, which is proportional to the number of Ga atoms, *N*_Ga_, in the respective GB phases (Equation 1). For the GB phase of pure Mg (0‐Ga), the absence of Ga makes the formation energy independent of µ_Ga_. This defect phase has the lowest energy at µ_Ga_ < −0.473 eV. As *N*_Ga_ increases in the 1‐Ga, 3‐Ga, and 6‐Ga phases, their formation energy decreases more sharply toward the Ga‐rich condition, so that they become the most stable phase at µ_Ga_ > −0.473 (1‐Ga), −0.353 (3‐Ga), and −0.190 eV (6‐Ga), respectively.

While local alloying enables us to interrogate a range of chemical potentials to explore new GB phases, the modeling of GB defect phase diagram provides the thermodynamic tool to understand defect phase transformations. As the experimental evaluation of chemical potentials is usually not straightforward, we elaborate on the interpretation and design of GB defect phase diagrams.

As shown in Figure 4, local alloying of Ga allows surveying the entire range of µ_Ga_ from the Ga‐free end to the Ga‐rich conditions. Due to the limited solubility of Ga in Mg (<1%, all numbers in atomic fractions), excessive amount of implanted Ga can build up a bulk‐phase layer of Ga on the sample surface, thus defining µ_Ga_ = 0 eV. At this µ_Ga_, we have indeed observed the predicted stable GB phase 6‐Ga (Figure 3a). However, it is worth noting that during the early stages of Ga incorporation (Figure 2), it is hard to relate the thermodynamic state to a specific µ_Ga_ corresponding to a local equilibrium.

Therefore, we propose to explore the lower chemical potentials as diffusion enables the transition from local states of thermodynamic equilibrium to more global states. There are several intermetallic phases in the Mg‐Ga phase diagram,^[^ ^68^ ^]^ so that direct interfacing of Mg with Ga is not the thermodynamic ground state. After sufficient time, the surface layer of Ga has diffused into the Mg matrix to form islands of Mg_5_Ga_2_ precipitates (Figure S7b–d, Supporting Information), the most Mg‐rich intermetallic phase in the Mg–Ga system.^[^ ^68^ ^]^

To evaluate µ_Ga_ in this condition, we notice that Ga atoms within the GB are in equilibrium with those in the Mg solid solution. For dilute Ga within the Mg solid solution, the chemical potential can be evaluated as µ_Ga_ = −0.222 eV + *k*_*B*_ *T*ln (*c*_Ga_), where −0.222 eV is the solution enthalpy of one Ga atom inside the Mg phase, and *k*_*B*_ *T*ln (*c*_Ga_) is the configurational entropy term at Ga concentration *c*_Ga_. Hence, µ_Ga_ is related to the local equilibrium with Ga solutes in Mg, as represented by the top axis of Figure 4.

This enables us to directly relate the computed defect phase diagram to our experimental observations. In particular, at the measured $c_{\mathrm{Ga}}=0.7\;\%$ in the solid solution (marked by an arrow in Figure 4), the experimentally observed 3‐Ga GB phase (Figure 3b) is indeed predicted to be the most stable. Furthermore, the evaluated transition from 0‐Ga (T‐type) and 1‐Ga (A‐type) µ_Ga_ = −0.473 eV corresponds to a very low Ga concentration of *c*_Ga_ = 6 · 10^−5^ at 300 K. Since this concentration is well within the solubility limit, there is no driving force to further lower µ_Ga_, and hence the A‐type structural units will remain stable for theΣ7 GB surrounded by Mg‐Ga solid solution.

### 2.5 Properties of GB Phases

Having established the thermodynamics of the GB phases in a defect phase diagram, their impact on the mechanical properties can be further studied. We start from the work of separation (WoS), a key parameter to predict the cohesion of GBs and hence the ductility of the materials. The computed WoS values for the defect phases are listed in the defect phase diagram (Figure 4), and the trend is plotted in Figure S8 (Supporting Information). Without Ga atoms, Mg Σ7 GBs have respectively 1.225 and 1.226 J m^−2^ WoS for T‐type and A‐type structures. The tiny difference suggests the phase transformation from T‐type to A‐type structural units plays a minor role in their integral mechanical properties. On the other hand, as shown in Figure S8, Ga segregation can lead to a significant increase in WoS. In other words, the presence of Ga atoms at Mg Σ7 GB phases enhances the resistance of GBs against decohesion and hence toughen the material. Up to the highest Ga numbers observed in an ordered GB phase, 6 atoms per structural unit, the increase in GB cohesion is monotonous, reaching 1.593 J m^−2^ (30% increase). Moreover, the increase from 0 to 3 Ga atoms per structural unit is almost linear, showing an enhancement up to 1.515 J m^−2^ (24% increase). As the 3‐Ga GB phase can stay in thermodynamic equilibrium with Mg–Ga solid solution, such toughening effect is readily applicable in Mg–Ga alloys.

Besides GB decohesion, segregation behavior at GBs can also change the GB mobility and hence influence the mechanical properties by different texture evolution and interaction with dislocations (Section 2.1). With advancing tools in multi‐scale modeling,^[^ ^26^ ^]^ phase diagrams have been established for GBs with increasingly complex geometry,^[^ ^79^, ^80^ ^]^ while mechanical properties can be modeled by molecular dynamics.^[^ ^61^ ^]^ With future access to machine‐learning enhanced Mg–Ga interatomic potentials for large‐ and multi‐scale modeling, we expect more in‐depth and holistic understanding between Ga segregation and mechanical properties can be gained.

## 3 Conclusion

The development of phase diagrams that predict the appearance of certain phases and structures as a function of temperature, pressure and chemical composition is one of the biggest success stories in materials science. It has helped to turn the empirical materials design approach into a knowledge‐based method that rests on strict thermodynamics. Here we have translated this principle to another essential property of materials, namely the lattice defects. We exemplified the design of a Mg–Ga alloy, which shows simultaneous improvement in tensile strength and ductility, owing to the segregation of Ga at GBs that enhances the cohesion at GBs and weakens the basal‐type texture.

We have examined different GB phases by combining atomic‐scale STEM characterization of defect structures, automatic pattern recognition and DFT modeling of their energetics. The chemical potential axis of the defect phase diagram is sampled experimentally by successive steps of local alloying as well as allowing time for diffusion to transit local thermodynamic equilibrium to a more global scale. We have driven and monitored the phase transformation of the same Mg Σ7 GB from a T‐type tetrahedron structural unit to A‐type capped trigonal prism unit by local alloying of Ga. Automatic pattern recognition algorithms have been developed to classify GB structural units and enabled tracing the defect phase transformation. Different GB phases with 6 and 3 columns of Ga atoms ordered in each structural unit have been identified using STEM, as their transformation was triggered by a shift in Ga chemical potential from local equilibrium with bulk Ga to the Mg–Ga solid solution. The experimental exploration covers a full range of chemical potentials relevant to the design of GB phases in Mg–Ga, and the structural information can be directly fed into atomistic modeling and narrows down the otherwise gargantuan space of configurations. DFT calculations not only provide the relative stability of different GB phases, but also connect them to local thermodynamic equilibrium by the chemical potential.

The experimentally‐initiated triggering of phase transformations and the systematic tracing of the same defects enable defect modeling without changing their geometry or boundary conditions, and facilitate straightforward construction of defect phase diagrams. The developed methodology can be generally applied to study many types of GBs and other lattice defects, provided they are accessible to high‐resolution structural and chemical imaging. For each defect, only one specimen with favorable imaging condition is necessary, as the local chemical potential of alloying elements can be tuned along and around the defect. We have demonstrated this combination of experiments and theory to expedite the construction of defect phase diagrams for their use in materials science and engineering.

## 4 Experimental Section

### Sample Preparation

Bulk samples of pure Mg and Mg‐0.7 at.% Ga were made from Mg (purity 99.95%) and Ga (purity 99.99%), molten and cast in an induction furnace at 730°C with an atmosphere of 500 mbar Ar and 50 mbar CO_2_. Both samples were then hot rolled to a total thickness reduction of 50% in steps of 10% thickness reduction at 430°C. Subsequently, the sheets were recrystallized at 450°C for 15 min followed by water quenching. Tensile test specimens were cut from the sheets by electrical discharge machining. Tensile tests were performed in the transverse direction (TD) using flat specimens with a gauge length of 10 mm and a constant cross‐section of 1.88 × 1.50 mm^2^. Tensile tests were performed under strain control on an electromechanical testing machine at an initial strain rate of 10^−3^ s^−1^. At least four specimens per material were tested.

The Mg thin film sample was sputter‐deposited onto an Ar plasma‐treated Si (100) substrate. Detailed description of the synthesis conditions is given in ref. [72], as well as the characterization of the sharp basal plane texture of Mg. The thin film hence contains numerous Mg [0001] tilt GBs with a random distribution of the misorientation angle.^[^ ^72^ ^]^ The Σ7 GBs were selected by examining GBs with a misorientation of ≈22°.

### Texture Analysis

Electron backscatter diffraction (EBSD) samples were metallographically prepared by mechanical grinding and polishing with diamond paste to 0.25 µm and then electropolished using Struers AC2. The Mg‐0.7 at.% Ga sample was additionally polished with OPS to obtain EBSD patterns. EBSD measurements were conducted on a Helios Nanolab 600i (Thermo Fisher) operated at 20 kV with pattern collection by a Hikari camera (EDAX Inc.). A D8 Advanced X‐ray goniometer (Bruker) was used to collect the pole figures. The samples were mechanically ground and polished to 3 µm with diamond paste. An area of at least 4 × 4 µm^2^ was measured on the ND‐TD plane. The halfwidth and resolution parameters used to calculate the orientation distribution function are both 3.5°.

### Local Alloying to Trigger Defect Phase Transformation

Focused ion beam (FIB) was applied to introduce the alloying element Ga locally into the area of examined GBs. First, the Ga‐free specimen for STEM was prepared on a plasma FIB (Thermo Fisher) starting with Xe^+^ polishing at 30 kV and ending with a cleaning step a 8 kV. The sample has a volume of 6.5 × 3 × 0.2 µm^3^, amounting to 280 fmol of Mg atoms. A Scios2 FIB (Thermo Fisher) was then employed to introduce Ga^+^ ions into the lamella. For that, cleaning cross‐section patterns were used with the sample tilted ±8° toward the Ga^+^ beam at 5 kV and 7.7 pA. In such condition, the sample receives 0.08 fmol of Ga^+^ ions for each second. Suppose an implantation rate of 100%, 1.7% of Ga relative to Mg would be introduced to the sample in a minute. From the measured Ga concentrations in the Mg sample, 0.50% and 1.24% after 1 min and 3 min Ga^+^ implantation, respectively, the implantation rates are evaluated as 29% (after 1 min) and 24% (after 3 min).

### Electron Microscopy

High‐resolution STEM imaging was performed on a Titan Themis microscope (Thermo Fisher) operated at 300 kV. Using an aberration‐corrected STEM probe of less than 0.1 nm size and 23.8 mrad convergence semi‐angle, STEM images were acquired using a HAADF detector with collection semi‐angles of 62‐200 mrad. EDS spectrum imaging was acquired using the SuperX detector. Multivariate statistical analysis was applied for noise reduction,^[^ ^81^ ^]^ and subsequent elemental quantification was performed using the Clif–Lorimer method. Precession electron diffraction 4D‐STEM imaging was performed on a JEM2200 microscope (JEOL) operated at 200 kV. The beam size was ≈2 nm and a precession angle of 0.5° was used.

### Computational Details

DFT calculations in this work have been carried out using the Vienna Ab initio Simulation Package (VASP)^[^ ^82^, ^83^ ^]^ with the projected augmented wave method^[^ ^84^ ^]^ to describe the interaction between ionic cores and valence electrons. The Perdew–Burke–Ernzerhof form of parameterization of the generalized gradient approximation has been used to describe the exchange‐correlation effects. A plane wave cutoff energy of 550 eV was used and based on the Monkhorst–Pack scheme,^[^ ^85^ ^]^ the Brillouin zone was sampled with a k‐point spacing of 0.12 nm^−1^ along all directions for all structures. The Methfessel–Paxton^[^ ^86^ ^]^ smearing scheme was applied with the smearing width set to 0.15 eV.

After the construction of supercells containing two GBs, they have been optimized by subjecting them to strains in the direction normal to the GB. A relaxation of atomic positions is performed in order to get to the equilibrium structure, thus preserving the lattice constants in the bulk regions of the supercell as well. The energy for this equilibrium structure is then applied to calculate the GB energy (γ_GB_) using the following equation:

(2) $$\gamma_{\mathrm{GB}}=\frac{E_{\mathrm{GB}}-E_{\mathrm{bulk}}}{2A_{\mathrm{GB}}}$$

where *E*_GB_ is the total energy of the supercell with the GBs, *E*_bulk_ is the energy of Mg bulk rescaled according to the number of atoms in the supercells for the GB and *A*_GB_ is the area of cross‐section of the GB. As each GB supercell contains two GBs, a factor of two is included in the denominator. To analyze the competition of the defect phases with and without Ga addition, we look into the formation energy *E*_f_ of the phases that are calculated using Equation (1), where *N* represents the number of Mg and Ga atoms in the supercell, and µ represents their chemical potentials.

The GB energy for each defect phase is used to compute the WoS which is given by,

(3) $$W_{\mathrm{sep}}=2\gamma_{\mathrm{surf}}-\gamma_{\mathrm{GB}}$$

where γ_surf_ is the surface formation energy. Similar to the GB energy, the surface formation energy is expressed as,

(4) $$\gamma_{\mathrm{surf}}=\frac{E_{\mathrm{surf}}-E_{\mathrm{bulk}}}{2A_{\mathrm{surf}}}$$

where E_surf_ is the total energy of the supercell containing the surfaces, A_surf_ is the area of cross‐section of the surface and as each supercell contains two surfaces the factor of two is included in the denominator.

### Automatic Pattern Recognition

To detect and distinguish the T‐type and A‐type structural units in STEM images, a novel mathematical framework was used that detects patterns in the form of atomic arrangements from simulations in atomic scale images and quantifies their differences by estimating the deviation between experimental and computational atomic arrangement. This framework uses a multi‐step procedure. First, the simulated atomic arrangement (“DFT structural units” in Figure S6, Supporting Information) is converted to a synthetic image patch with a sum of Gaussians centered at the simulated positions. The potential occurrences of this patch in the STEM image are found with template matching using normalized correlation (see “Experimental structural units” for example image patch matches in Figure S6, Supporting Information). On each matching patch, the deviation between the simulated atomic column arrangement to the positions in the STEM image is estimated using bump fitting (“Atomic position matching” exemplifies the deviation as shift for each atom in the structural unit in Figure S6, Supporting Information). The fitting of the positions from the simulated pattern to the experimental image is split into two steps: First the affine part of the deviation is determined, then the remaining nonlinear part.

From the resulting fit, feature descriptors that allow to determine which of the given patterns is present were derived. Noting that near a candidate location for an A‐type match, there are two neighboring candidates for a T‐type match. The same descriptors on these neighboring positions were derived using the T‐type arrangement. Only one of the two neighboring matches is reasonable, as the other one can be easily determined from the large deviation necessary to fit the simulation pattern to the image and is discarded (Figure S6, Supporting Information shows only the reasonable match). Thus, for each A‐type match, two sets of descriptors were used, one describing how well the A‐type fits, the other one on the T‐type fits.

Three descriptors are employed for the decision making: 1. The standard deviation of the horizontal coordinates of the three atomic sites **a1**, **a2**, and **a3** (see Figure S2, Supporting Information for the atomic site labels). A number close to zero corresponds to a straight GB plane; 2. The absolute difference in *y* coordinates between the **b1** and **b2** sites; 3. The absolute difference in *y* coordinates between the **d1** and **d2** sites. For the latter two descriptors, a number close to zero corresponds to a better mirror symmetry with respect to the GB plane. For a perfect T‐type structure, all three descriptors have zero values, while the neighboring A‐type motifs return bigger values and can be excluded. Likewise, for a perfect A‐type structure, all three descriptors are zero, while the neighboring T‐type motifs return bigger values and can be excluded. For decision making on the experimental images, the structural units with two or all of three descriptors returning smaller values (closer to zero) are designated (“Decision making” in Figure S6, Supporting Information).

To visualize entire STEM image as a grid topology, the matched GB structural units are patched with atomic columns of the neighboring grains. For this purpose, the motif extraction approach described in ref. [87] was applied to determine the unit cell motifs of the left and right grains in the STEM images. The hexagons from both grains are then constructed and merged with the four‐sided polygons from the detected T‐type units and the eight‐sided polygons from the detected A‐type units to describe the atomic grid topology.

### STEM Multi‐Slice Image Simulation

The purpose of the image simulation is to generate simulated HAADF images based on the DFT calculated structures and compare them with the experimental measurements. The STEM multi‐slice simulations were performed using the muSTEM (v5.2) software package.^[^ ^88^ ^]^ The microscope parameters for the simulations, such as the half‐convergence angle (23.8 mard), primary electron energy (300 kV), and HAADF detector (62‐200 mrad), were chosen according to the experimental conditions. Figure S9 (Supporting Information) displays the multislice STEM simulations of the structural models derived from DFT calculations. These models represent the T‐type structural unit of pure Mg Σ7 GB, as well as the A‐type units with 3‐atom and 6‐atom configurations.

## Conflict of Interest

The authors declare no conflict of interest.

## Supporting information

Supporting Information

## Data Availability

The data that support the findings of this study are available in the supplementary material of this article.

## References

[1] C. C. Tasan , M. Diehl , D. Yan , M. Bechtold , F. Roters , L. Schemmann , C. Zheng , N. Peranio , D. Ponge , M. Koyama , K. Tsuzaki , D. Raabe , Annu. Rev. Mater. Res. 2015, 45, 391.

[2] A. Sharma , V. V. Tyagi , C. R. Chen , D. Buddhi , Renewable Sustainable Energy Rev. 2009, 13, 318.

[3] D. Raabe , B. Sander , M. Friák , D. Ma , J. Neugebauer , Acta Mater. 2007, 55, 4475.

[4] J. W. Gibbs , The Collected Works of J. Willard Gibbs., Technical report, Yale Univ. Press, New Haven 1948.

[5] J. Buban , K. Matsunaga , J. Chen , N. Shibata , W. Ching , T. Yamamoto , Y. Ikuhara , Science 2006, 311, 212. 16410521 10.1126/science.1119839

[6] L. Lu , M. Sui , K. Lu , Science 2000, 287, 1463. 10688789 10.1126/science.287.5457.1463

[7] M. Legros , G. Dehm , E. Arzt , T. J. Balk , Science 2008, 319, 1646. 18356520 10.1126/science.1151771

[8] L. Lu , Y. Shen , X. Chen , L. Qian , K. Lu , Science 2004, 304, 422. 15031435 10.1126/science.1092905

[9] A. H. Cottrell , B. A. Bilby , Proc. Phys. Soc. Sect. A 1949, 62, 49.

[10] M. Kuzmina , M. Herbig , D. Ponge , S. Sandlöbes , D. Raabe , Science 2015, 349, 1080. 26339026 10.1126/science.aab2633

[11] X. Zhou , J. R. Mianroodi , A. Kwiatkowski da Silva , T. Koenig , G. B. Thompson , P. Shanthraj , D. Ponge , B. Gault , B. Svendsen , D. Raabe , Sci. Adv. 2021, 7, eabf0563. 33863726 10.1126/sciadv.abf0563 PMC8051869

[12] Y. Yu , C. Zhou , X. Zhang , L. Abdellaoui , C. Doberstein , B. Berkels , B. Ge , G. Qiao , C. Scheu , M. Wuttig , O. Cojocaru‐Mirédin , S. Zhang , Nano Energy 2022, 101, 107576.

[13] H. Suzuki , J. Phys. Soc. Jpn. 1962, 17, 322.

[14] D. Palanisamy , D. Raabe , B. Gault , Acta Mater. 2019, 174, 227.

[15] P. Lejček , S. Hofmann , Acta Metall. Mater. 1991, 39, 2469.

[16] L.‐S. Chang , E. Rabkin , B. Straumal , B. Baretzky , W. Gust , Acta Mater. 1999, 47, 4041.

[17] R. Kirchheim , Acta Mater. 2002, 50, 413.

[18] Z. Wang , M. Saito , K. P. McKenna , L. Gu , S. Tsukimoto , A. L. Shluger , Y. Ikuhara , Nature 2011, 479, 380. 22094698 10.1038/nature10593

[19] A. Rečnik , S. Bernik , N. Daneu , J. Mater. Sci. 2012, 47, 1655.

[20] T. Chookajorn , H. A. Murdoch , C. A. Schuh , Science 2012, 337, 951. 22923577 10.1126/science.1224737

[21] J. F. Nie , Y. Zhu , J. Liu , X.‐Y. Fang , Science 2013, 340, 957. 23704567 10.1126/science.1229369

[22] D. Raabe , M. Herbig , S. Sandlöbes , Y. Li , D. Tytko , M. Kuzmina , D. Ponge , P. P. Choi , Curr. Opin. Solid State Mater. Sci. 2014, 18, 253.

[23] Z. Yu , P. R. Cantwell , Q. Gao , D. Yin , Y. Zhang , N. Zhou , G. S. Rohrer , M. Widom , J. Luo , M. P. Harmer , Science 2017, 358, 97. 28983049 10.1126/science.aam8256

[24] P. Lejček , M. Šob , V. Paidar , Prog. Mater. Sci. 2017, 87, 83.

[25] X. Zhou , A. Ahmadian , B. Gault , C. Ophus , C. H. Liebscher , G. Dehm , D. Raabe , Nat. Commun. 2023, 14, 3535. 37316498 10.1038/s41467-023-39302-x PMC10267137

[26] C. Hu , R. Dingreville , B. L. Boyce , Comput. Mater. Sci. 2024, 232, 112596.

[27] E. W. Hart , Scripta Metall. 1968, 2, 179.

[28] T. Frolov , Y. Mishin , J. Chem. Phys. 2015, 143, 044706. 26233156 10.1063/1.4927414

[29] T. Brink , L. Langenohl , H. Bishara , G. Dehm , Phys. Rev. B: Condens. Matter Mater. Phys. 2022, 107, 054103.

[30] S. J. Dillon , M. Tang , W. C. Carter , M. P. Harmer , Acta Mater. 2007, 55, 6208.

[31] M. P. Harmer , Science 2011, 332, 182. 21474743 10.1126/science.1204204

[32] J. Luo , H. Cheng , K. M. Asl , C. J. Kiely , M. P. Harmer , Science 2011, 333, 1730. 21940889 10.1126/science.1208774

[33] W. D. Kaplan , D. Chatain , P. Wynblatt , W. C. Carter , J. Mater. Sci. 2013, 48, 5681.

[34] P. R. Cantwell , M. Tang , S. J. Dillon , J. Luo , G. S. Rohrer , M. P. Harmer , Acta Mater. 2014, 62, 1.

[35] S. Johansson , G. Wahnström , Phys. Rev. B: Condens. Matter Mater. Phys. 2012, 86, 035403.

[36] K.‐D. Bauer , M. Todorova , K. Hingerl , J. Neugebauer , Acta Mater. 2015, 90, 69.

[37] D. Scheiber , M. N. Popov , P. Supancic , J. Spitaler , Acta Mater. 2022, 229, 117804.

[38] S. Korte‐Kerzel , T. Hickel , L. Huber , D. Raabe , S. Sandlöbes‐Haut , M. Todorova , J. Neugebauer , Int. Mater. Rev. 2022, 67, 89.

[39] H. Bishara , S. Lee , T. Brink , M. Ghidelli , G. Dehm , ACS Nano 2021, 15, 16607. 34605639 10.1021/acsnano.1c06367 PMC8552493

[40] R. Bueno Villoro , D. Zavanelli , C. Jung , D. A. Mattlat , R. Hatami Naderloo , N. Pérez , K. Nielsch , G. J. Snyder , C. Scheu , R. He , S. Zhang , Adv. Energy Mater. 2023, 13, 2204321.

[41] R. Bueno Villoro , M. Wood , T. Luo , H. Bishara , L. Abdellaoui , D. Zavanelli , B. Gault , G. J. Snyder , C. Scheu , S. Zhang , Acta Mater. 2023, 249, 118816.

[42] M. Duerrschnabel , M. Yi , K. Uestuener , M. Liesegang , M. Katter , H. J. Kleebe , B. Xu , O. Gutfleisch , L. Molina‐Luna , Nat. Commun. 2017, 8, 1. 28676636 10.1038/s41467-017-00059-9 PMC5496909

[43] E. O. Hall , Proc. Phys. Soc. Sect. B 1951, 64, 747.

[44] R. Wu , A. Freeman , G. B. Olson , Science 1994, 265, 376. 17838041 10.1126/science.265.5170.376

[45] A. Khalajhedayati , Z. Pan , T. J. Rupert , Nat. Commun. 2016, 7, 10802. 26887444 10.1038/ncomms10802 PMC4759628

[46] A. R. Krause , P. R. Cantwell , C. J. Marvel , C. Compson , J. M. Rickman , M. P. Harmer , J. Am. Ceram. Soc. 2018, 102, 778.

[47] P. R. Cantwell , T. Frolov , T. J. Rupert , A. R. Krause , C. J. Marvel , G. S. Rohrer , J. M. Rickman , M. P. Harmer , Annu. Rev. Mater. Res. 2020, 50, 465.

[48] G. Dehm , J. Cairney , MRS Bull. 2022, 47, 800.

[49] J. W. Cahn , J. Phys. Colloques 1982, 43, C6.

[50] C. Rottman , MRS Proc. 1991, 238, 191.

[51] M. Tang , W. C. Carter , R. M. Cannon , Phys. Rev. B 2006, 73, 024102. 10.1103/PhysRevLett.97.075502 17026243

[52] T. Frolov , Y. Mishin , Phys. Rev. B: Condens. Matter Mater. Phys. 2012, 85, 12.

[53] T. Frolov , Y. Mishin , Phys. Rev. B: Condens. Matter Mater. Phys. 2012, 85, 22.

[54] T. Frolov , S. V. Divinski , M. Asta , Y. Mishin , Phys. Rev. Lett. 2013, 110, 1. 10.1103/PhysRevLett.110.255502 23829744

[55] T. Meiners , T. Frolov , R. E. Rudd , G. Dehm , C. H. Liebscher , Nature 2020, 579, 375. 32188953 10.1038/s41586-020-2082-6 PMC7100613

[56] L. Frommeyer , T. Brink , R. Freitas , T. Frolov , G. Dehm , C. H. Liebscher , Nat. Commun. 2022, 13, 1. 35680878 10.1038/s41467-022-30922-3 PMC9184537

[57] T. G. Ference , R. W. Balluffi , Scripta Metall. 1988, 22, 1929.

[58] N. J. Peter , M. J. Duarte , C. Kirchlechner , C. H. Liebscher , G. Dehm , Acta Mater. 2021, 214, 116960.

[59] H. Zhao , P. Chakraborty , D. Ponge , T. Hickel , B. Sun , C.‐H. Wu , B. Gault , D. Raabe , Nature 2022, 602, 437. 35173345 10.1038/s41586-021-04343-z PMC8850197

[60] W. Sigle , G. Richter , M. Rühle , S. Schmidt , Appl. Phys. Lett. 2006, 89, 1.

[61] C. Hu , Y. Li , Z. Yu , J. Luo , npj Comput. Mater. 2021, 7, 159.

[62] H. B. Liu , G. H. Qi , Y. T. Ma , H. Hao , F. Jia , S. H. Ji , H. Y. Zhang , X. G. Zhang , Mater. Sci. Eng., A 2009, 526, 7.

[63] J. Kubásek , D. Vojtiěch , Int. J. Mater. Res. 2016, 107, 459.

[64] Z. Gao , M. Song , R. L. Liu , Y. Shen , L. Ward , I. Cole , X. B. Chen , X. Liu , Mater. Sci. Eng., C 2019, 104, 109926. 10.1016/j.msec.2019.109926 31499938

[65] W. Huang , J. Chen , H. Yan , W. Xia , B. Su , H. Yin , X. Yan , J. Mater. Sci. 2020, 55, 10242.

[66] D. He , Y. Li , Y. Zheng , X. Yue , Y. Wu , X. Xue , H. Yu , W. Li , Y. Li , J. Alloys Compd. 2021, 887, 161317.

[67] W. Huang , J. Chen , H. Yan , Q. Li , W. Xia , B. Su , W. Zhu , Trans. Nonferrous Met. Soc. China (Engl. Ed.) 2022, 32, 2852.

[68] Y. Feng , R. Wang , H. Liu , Z. Jin , J. Alloys Compd. 2009, 486, 581.

[69] J. Wu , L. Jin , J. Dong , F. Wang , S. Dong , J. Mater. Sci. Technol. 2020, 42, 175.

[70] T. Watanabe , J. Mater. Sci. 2011, 46, 4095.

[71] M. R. Barnett , Metall. Mater. Trans. A 2003, 34 A, 1799.

[72] S. Zhang , Z. Xie , P. Keuter , S. Ahmad , L. Abdellaoui , X. Zhou , N. Cautaerts , B. Breitbach , S. Aliramaji , S. Korte‐Kerzel , M. Hans , J. M. Schneider , C. Scheu , Nanoscale 2022. 10.1039/d2nr05505h 36454106

[73] Y. C. Wang , H. Q. Ye , Phil. Mag. A: Phys. Condens. Matter, Struct., Defects Mech. Prop. 1997, 75, 261.

[74] M. Ashby , F. Spaepen , S. Williams , Acta Metall. 1978, 26, 1647.

[75] R. C. Pond , V. Vitek , D. A. Smith , Acta Crystallogr. A 1979, 35, 689.

[76] A. P. Sutton , Philos. Mag. Lett. 1989, 59, 53.

[77] L. Huber , J. Rottler , M. Militzer , Acta Mater. 2014, 80, 194.

[78] Y. Sato , T. Yamamoto , Y. Ikuhara , J. Am. Ceram. Soc. 2007, 90, 337.

[79] C. Hu , J. Luo , Scr. Mater. 2019, 158, 11.

[80] C. Hu , Y. Zuo , C. Chen , S. Ping Ong , J. Luo , Mater. Today 2020, 38, 49.

[81] S. Zhang , C. Scheu , Microscopy 2018, 67, i133. 29136225 10.1093/jmicro/dfx091 PMC7207561

[82] G. Kresse , J. Hafner , Phys. Rev. B 1993, 47, 558. 10.1103/physrevb.47.558 10004490

[83] G. Kresse , J. Furthmüller , Phys. Rev. B 1996, 54, 11169. 10.1103/physrevb.54.11169 9984901

[84] G. Kresse , D. Joubert , Phys. Rev. B 1999, 59, 1758.

[85] H. J. Monkhorst , J. D. Pack , Phys. Rev. B 1976, 13, 5188.

[86] M. Methfessel , A. T. Paxton , Phys. Rev. B 1989, 40, 3616. 10.1103/physrevb.40.3616 9992329

[87] A. S. A. Alhasan , S. Zhang , B. Berkels , Ultramicroscopy 2023, 254, 113827. 37716773 10.1016/j.ultramic.2023.113827

[88] L. Allen , A. D'Alfonso , S. Findlay , Ultramicroscopy 2015, 151, 11. 25467859 10.1016/j.ultramic.2014.10.011

