# Supplementary material for: Materials Design by Constructing Phase Diagrams for Defects
Source: Adv Mater. 2024 Nov 17;37(3):2402191. doi: 10.1002/adma.202402191 (PMC11756050; doi:10.1002/adma.202402191)
Supplement: Supplementary file 1 — Supporting Information [file ADMA-37-2402191-s001.pdf]

ADVANCED MATERIALS

Supporting Information

for *Adv. Mater.*, DOI 10.1002/adma.202402191

Materials Design by Constructing Phase Diagrams for Defects

Xuyang Zhou, Prince Mathews, Benjamin Berkels, Wassilios Delis, Saba Saood, Amel Shamseldeen Ali Alhassan, Philipp Keuter, Jochen M. Schneider, Sandra Korte-Kerzel, Stefanie Sandlöbes-Haut, Dierk Raabe, Jörg Neugebauer, Gerhard Dehm, Tilmann Hickel*, Christina Scheu* and Siyuan Zhang**

A Supplemental Note S1

According to the Brandon's criterion [D. G. Brandon, Acta Metall. 1966, 14, 1479], $\Sigma 7$ [0001] tilt GBs are characterized by a large range of misorientation angles around $\approx 22°$, up to a deviation of $15°/\sqrt{7} = 5.7°$. Using this criterion, although many GBs are classified as $\Sigma 7$, we choose GBs that are very close to 22° (±1°) to fit closely with atomistic modelling, as well as those with flat GB planes edge-on to the viewing direction to facilitate atomic resolution imaging.
We managed to capture a near- $\Sigma 7$ [0001] tilt GB with 19° misorientation after 1 day of Ga FIB preparation. The Gibbsian interfacial excess was evaluated as $\Gamma_{Ga} = 11.8$ Ga/nm^2, corresponding to 5.2 Ga/$\Sigma 7$ unit. Using the low angle grain boundary model suggested in Ref. [72], Γ_{Ga} at $\Sigma 7$ CSL angle (22°) can be further evaluated as $\Gamma_{Ga} = 11.8 \sin(22°/2)/\sin(19°/2)$ Ga/nm^2 = 13.6 Ga/nm^2 = 6.0 Ga/$\Sigma 7$ unit.

B Supplemental Figures

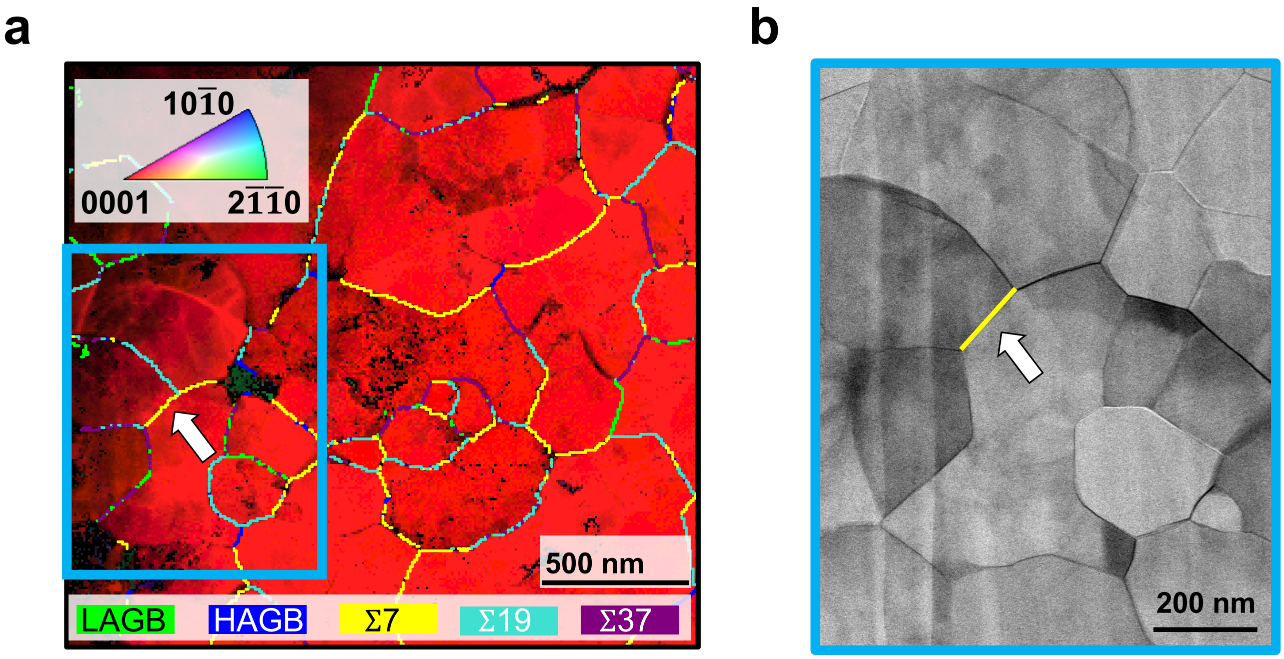

Figure S1: (a) Orientation and GB maps reconstructed from the 4D-STEM data set. The thin film sample shows sharp (0001) texture (red color). Grains with a confidence index of less than 0.1 are shown in black. (b) Bright-field STEM image for the highlighted region in (a). White arrows in both figures point to the $\Sigma 7$ GB for the high-resolution STEM study.

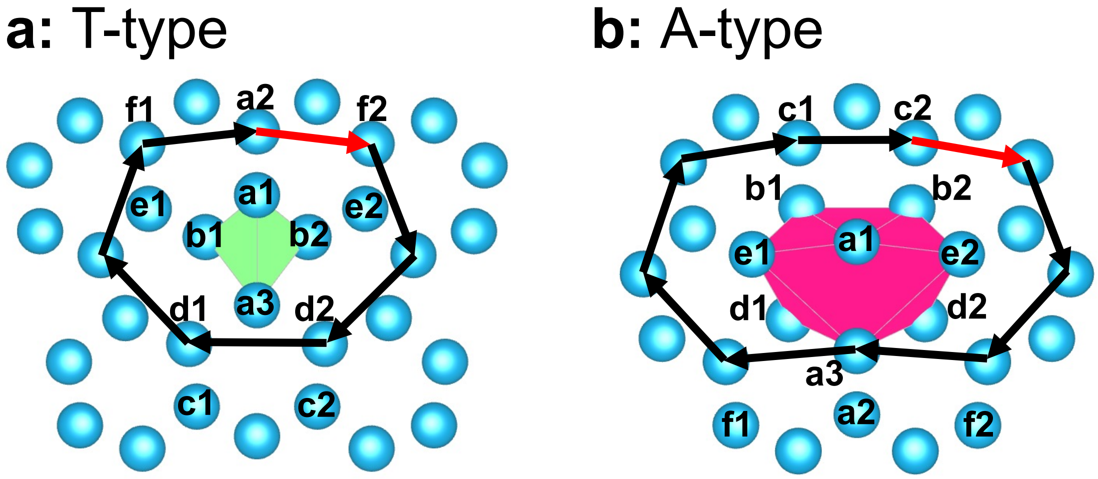

Figure S2: Burgers circuit analysis for the (a) T-type and (b) A-type structural units. The black arrows show pairs of $\frac{1}{3}\langle 2\overline{1}\overline{1}0\rangle$ vectors that are closed by the Burgers vectors $\vec{b} = \frac{1}{3}[2\overline{1}\overline{1}0]$ (red arrows). The nomenclature for the atomic columns is shown on top of them.

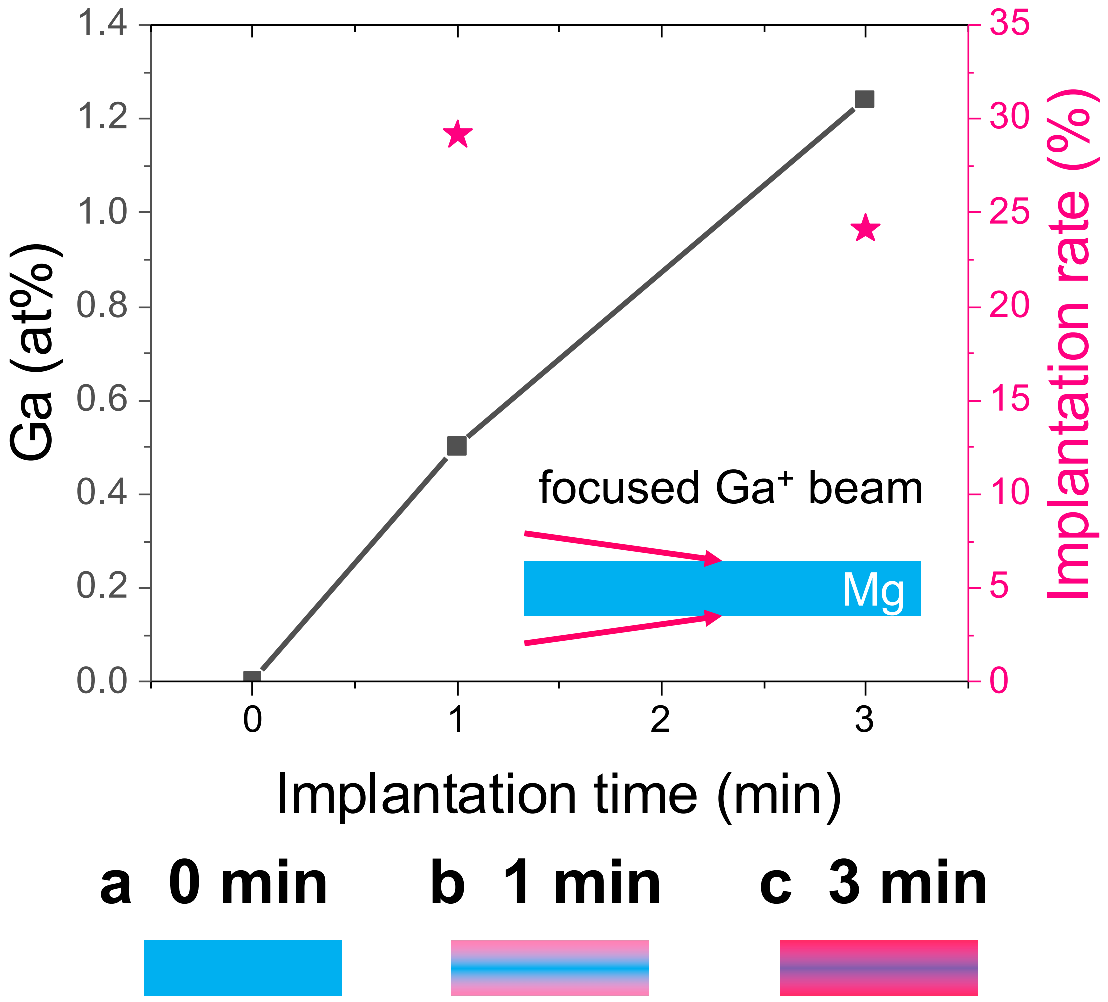

Figure S3: The Ga composition inside the Mg sample as a function of the implantation time and the evaluated implantation rate. Schematics of Ga^+ implantation by FIB is displayed in the inset at 5 kV, 7.7 pA, and grazing incidence of ±8°. The three sample states of (a) pure Mg, after (b) 1 min and (c) 3 min implantation are schematically shown.

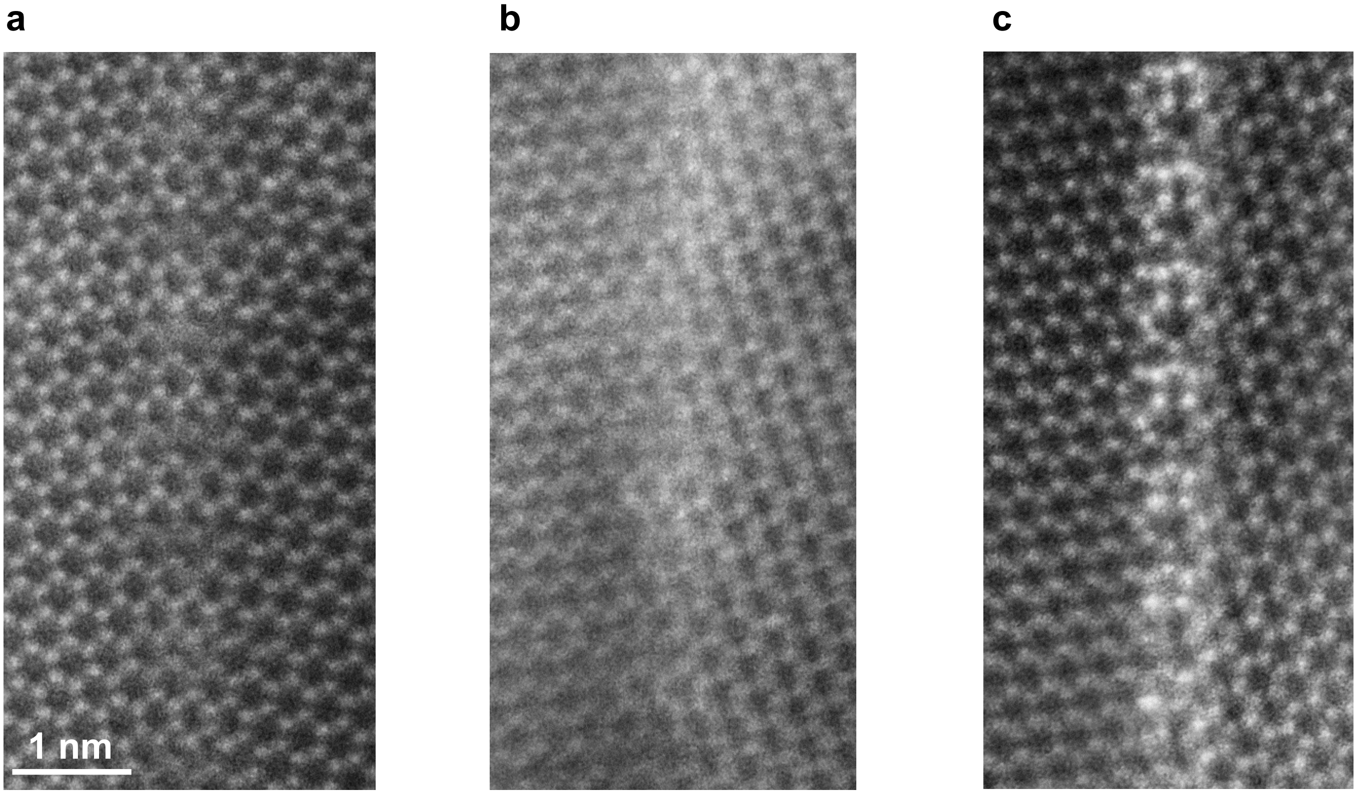

Figure S4: HAADF-STEM images without overlaid grids, corresponding to the ones presented in Fig. 2.

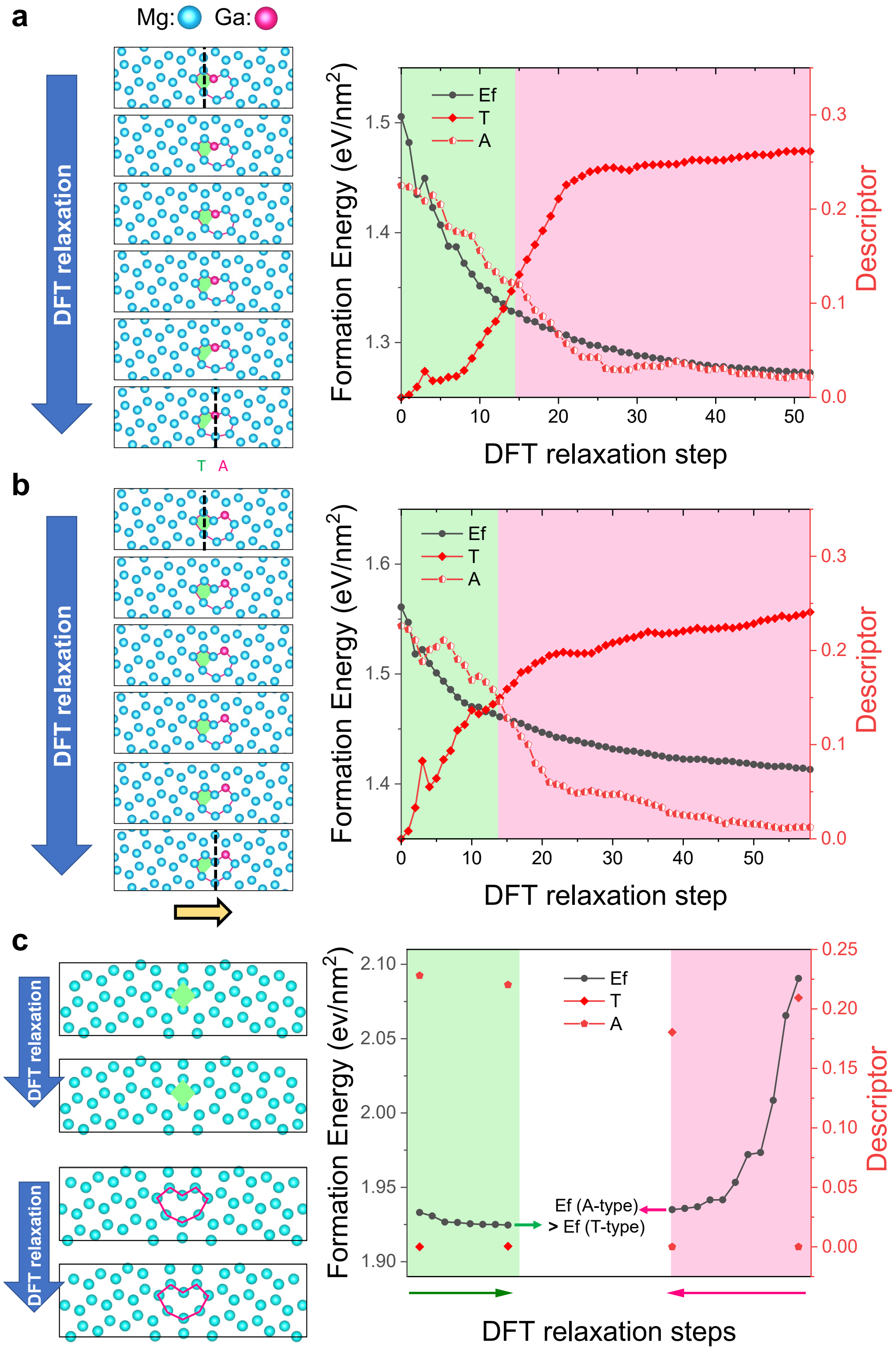

Figure S5: Snapshots of DFT structural relaxation starting with a T-type unit with Ga atoms on the (a) **b2** and (b) **e2** sites, ending to an A-type unit with Ga atoms on the (a) **a1** and (b) **b2** sites. (c) DFT structural relaxation starting with either T-type or A-type unit without Ga atoms in the supercell keeps their respectively GB structural unit. The left column of each image emphasizes the atomic structure at GBs, with the structural units of T-type and A-type distinguished by solid green shading and pink outline, respectively. The shift in GB plane is indicated by the arrow. On the right column, the images display the evolution of formation energy as DFT relaxation steps. The descriptors to match T-type and A-type structural units are plotted in the same diagram. As a perfect match corresponds to a descriptor value of 0, the T-type and A-type structures are classified according to the lower value of their descriptors and shaded in green and pink colors, respectively.

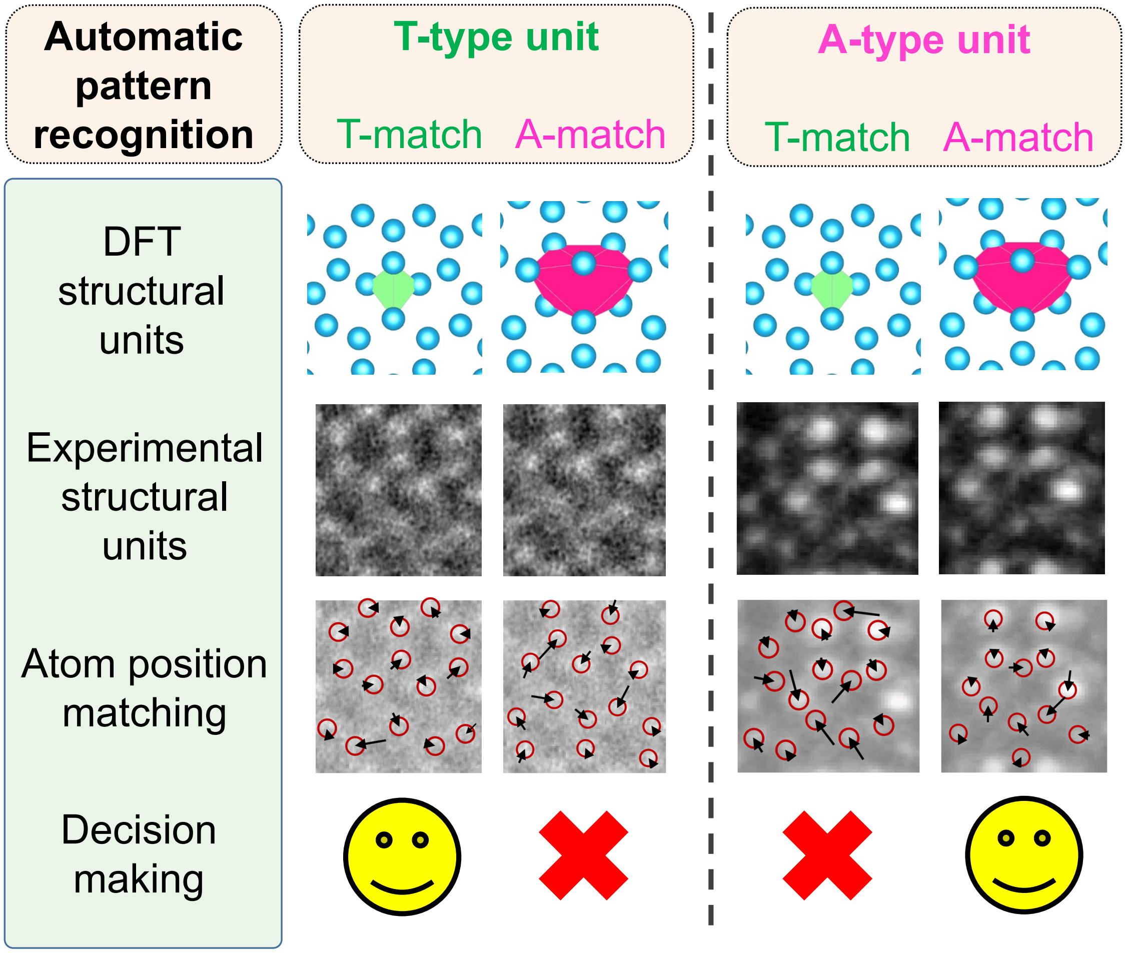

Figure S6: **Automatic pattern recognition to classify experimental images into T-type and A-type structural units.** The displacement vectors between DFT and experimental structures are magnified by five times for visualization.

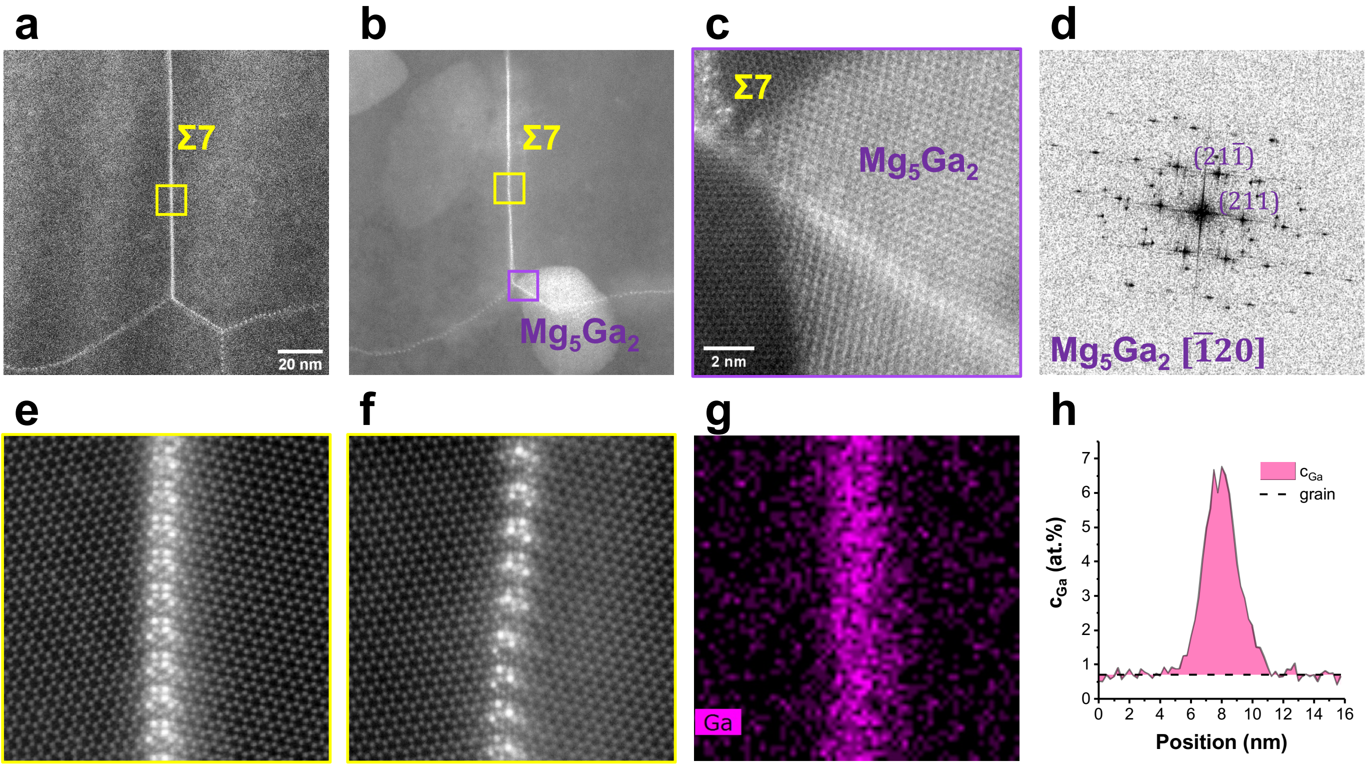

Figure S7: HAADF-STEM images of the same Σ7 GB (a) 1 day and (b) 620 days after Ga^+ beam thinning. The long storage time enabled longer range Ga diffusion to form bulk precipitates characterized as Mg_5Ga_2 by (c) high resolution HAADF-STEM image and (d) the corresponding fast Fourier transformation. (e,f) High resolution HAADF-STEM images of the same Σ7 GB (e) 1 day and (f) 620 days after Ga^+ beam thinning. (g) EDS Ga maps of the corresponding area in (f) and (h) Ga composition profile across the GB.

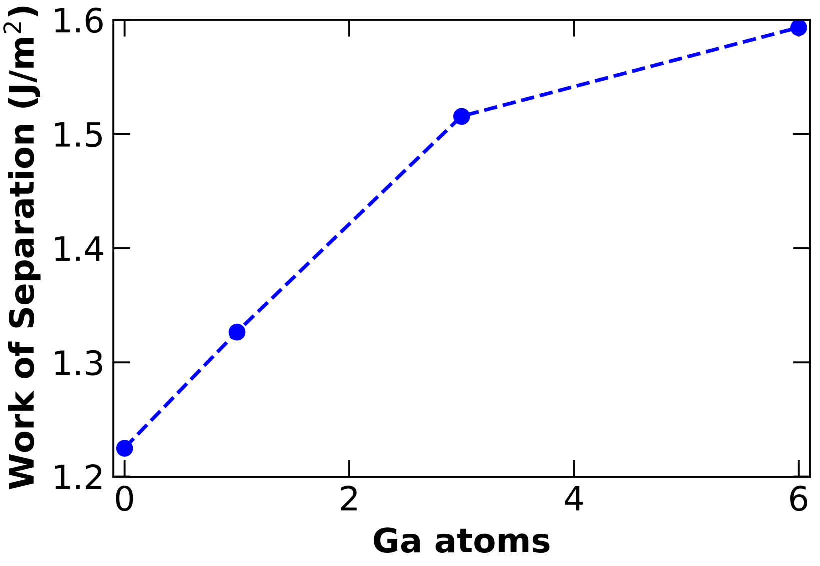

Figure S8: **WoS for the defect phases of the Mg Σ7 GB.** The calculated WoS for the experimentally observed defect phases of Mg Σ7 GB using DFT. The bottom axis represents the number of Ga atoms per unit cell of the defect phase.

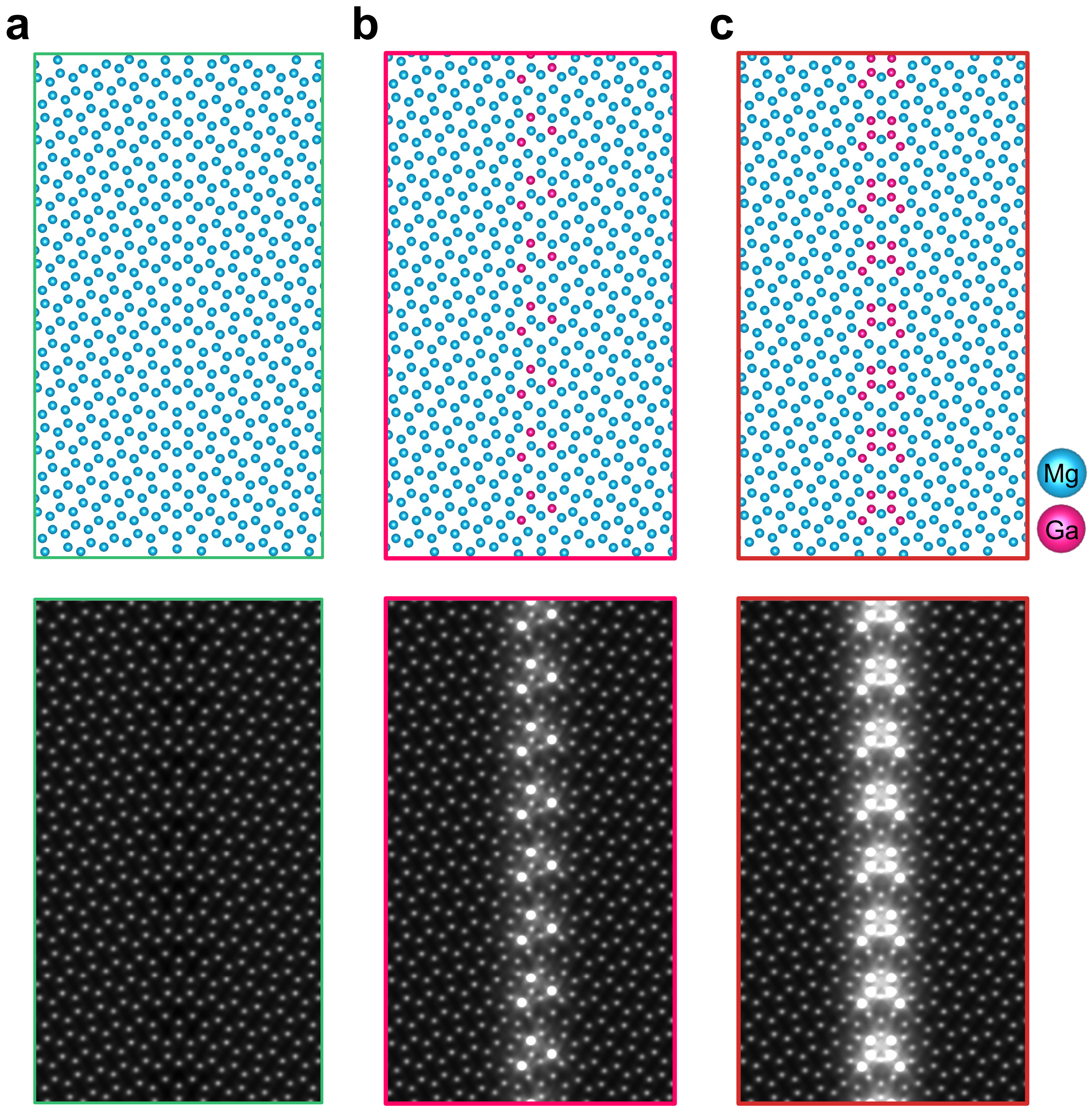

Figure S9: Multislice STEM simulations for the structural models obtained from DFT calculations. Top: atomistic structural model. Bottom: Multislice STEM simulations. (a) T-type pure Mg Σ7 GB, and A-type units with (b) three and (c) six Ga columns.
